# Identification and Characterization of Three Novel Lipases Belonging to Families II and V from *Anaerovibrio lipolyticus* 5ST

**DOI:** 10.1371/journal.pone.0069076

**Published:** 2013-08-12

**Authors:** Florence Privé, Naheed N. Kaderbhai, Susan Girdwood, Hilary J. Worgan, Eric Pinloche, Nigel D. Scollan, Sharon A. Huws, C. Jamie Newbold

**Affiliations:** Institute of Biological, Environmental and Rural Sciences, Aberystwyth University, Aberystwyth, Ceredigion, United Kingdom; Columbia University, United States of America

## Abstract

Following the isolation, cultivation and characterization of the rumen bacterium *Anaerovibrio lipolyticus* in the 1960s, it has been recognized as one of the major species involved in lipid hydrolysis in ruminant animals. However, there has been limited characterization of the lipases from the bacterium, despite the importance of understanding lipolysis and its impact on subsequent biohydrogenation of polyunsaturated fatty acids by rumen microbes. This study describes the draft genome of *Anaerovibrio lipolytica* 5ST, and the characterization of three lipolytic genes and their translated protein. The uncompleted draft genome was 2.83 Mbp and comprised of 2,673 coding sequences with a G+C content of 43.3%. Three putative lipase genes, *alipA*, *alipB* and *alipC*, encoding 492-, 438- and 248- amino acid peptides respectively, were identified using RAST. Phylogenetic analysis indicated that alipA and alipB clustered with the GDSL/SGNH family II, and alipC clustered with lipolytic enzymes from family V. Subsequent expression and purification of the enzymes showed that they were thermally unstable and had higher activities at neutral to alkaline pH. Substrate specificity assays indicated that the enzymes had higher hydrolytic activity against caprylate (C8), laurate (C12) and myristate (C14).

## Introduction

Hobson and Mann [1] isolated a bacterium from the sheep rumen able to hydrolyze linseed oil triglycerides to glycerol and fatty acids, using anaerobic techniques and a combination of differential and selective media [2]. It was named *Anaerovibrio lipolytica* [3], since changed to *A. lipolyticus* [4]. The growth characteristics of strain 5ST were described in continuous culture. Ribose, fructose and D-lactate were used as growth substrates and glycerol was fermented to propionate, lactate and succinate [5], [6], [7]. Ruminal lipase activity in animals receiving mainly concentrate feeds is thought to be accomplished mainly by *A. lipolyticus*, although other lipolytic species might be expected to predominate in grazing animals as *A. lipolyticus* lacks the ability to hydrolyze galacto- and phospholipids [8]. These latter lipids are known to be hydrolyzed in vitro by *Butyrivibrio fibrisolvens* strains S2 and LM8/1B [9]. Culture studies have shown *A. lipolyticus* to be present at around 10^7^/ml in rumen [10] and molecular studies based on the concentration 16S rDNA have tended to support this [11], [12], [13] suggesting that a major role for *A. lipolyticus* in the rumen. Despite the possible importance of *A. lipolyticus* in ruminal lipid metabolism its lipase activity remains relatively unstudied. *A. lipolyticus* extracellular lipase activity was characterized in cell free-medium and after purification by chromatography on Sephadex columns; the lipases were most active at pH 7.4 and 20 to 22°C, and diglycerides were hydrolyzed more rapidly than triglycerides [8], [14], [15]. However no recent studies have been undertaken to enhance our knowledge of the lipases in *A. lipolyticus*. This study describes a genomic analysis of *A. lipolyticus* 5ST using the 454 pyrosequencing technology (Roche, Life Sciences) and the identification of three lipolytic genes in this important rumen organism; their expression and the subsequent purification and characterization of the protein products from these genes.

## Materials and Methods

### Preparation of Anaerovibrio lipolyticus 5ST genomic DNA

Pure cultures of *A. lipolyticus* strain 5ST, as first isolated by Hobson and Mann [1] at the Rowett Research Institute (Aberdeen, Scotland), came from the Herbivore Gut Ecosystems group collection at IBERS. The genomic DNA was extracted using the BIO101 FastDNA® Spin Kit for Soil (Qbiogene, Cambridge, UK) from approximately 2 mg of cryopreserved freeze-dried culture. The manufacturer's guidelines were followed, with the exception that the sample was processed for 3×30 s at speed 6.0 in the FastPrep instrument (QBiogene), with incubation for 30 s on ice between bead-beating.

### De novo genome sequencing

The draft nucleotide sequence of the bacterium was established by a shotgun sequencing approach carried out on a Genome Sequencer FLX system (454 Life Sciences, Roche), following the supplier's protocol. Assembly of the reads was accomplished using gsAssembler v2.5.3 software (Roche, Life Sciences), using the default parameters.

### Annotation and sequence analysis

The contigs were submitted for genome annotation to the RAST server at http://rast.nmpdr.org [16], tRNAscan-SE 1.23 [17] and RNAmmer 1.2 [18].

The predicted lipase genes and amino acid sequences were compared for similarity to known sequences using BLASTN and BLASTP search. Their signal sequences for peptide cleavage were predicted using SignalP 4.0 [19]. CD search [20], the Pfam database (version 25.0, available at http://pfam.sanger.ac.uk/) and ClustalW [21] were used to search for conserved domains in the predicted amino acid sequences and to execute multiple alignments to find potential gene products relatedness to known families of lipolytic enzymes. The theoretical molecular mass and isoelectric point of the deduced lipolytic protein sequences were calculated using the Compute pI/Mw tool on the ExPASy proteomics server (available at http://expasy.org/tools/pi_tool.html, May 2011).

### Expression and purification of recombinant lipases

Primers for the amplification of the lipase genes were designed with FastPCR 6.1 [22], with and without the N-terminal signal sequence where one could be identified (Table S2). The PCR reaction was set up in a total volume of 25 µl as follows: 2 µL of template (∼100 ng), 1 µl of forward and reverse primer (10 pM), 8.5 µl of molecular water and 12.5 µl of PCR mastermix (ImmoMix™, Bioline UK Ltd., London, UK). Initial activation of the *Taq* was performed for 10 min at 95°C, followed by 25 cycles as follows: 95°C for 30 s, 50°C for 30 s, 72° for 2 min, followed by a final extension at 72°C for 8 min and holding of samples at 4°C. After PCR, the products were verified by electrophoresis on a 1% agarose gel using a 1 kb ladder. The band of interest was cut out with a sterile razor blade and the DNA eluted using the MinElute Gel Extraction kit (Qiagen, Crawley, UK).

The expression of the lipolytic genes was then undertaken using the pTrcHis TOPO® TA Expression kit (Invitrogen, Carlsbad, CA, USA) following the supplier's protocol. The PCR product was ligated to the pTrcHis TOPO vector and introduced into *E. coli* TOP10 cells. Twelve colonies for each transformation were picked for secondary screening and their insert was analysed for size and orientation by tip-dip PCR using the gene specific forward primer and the vector specific pTrcHis reverse primer (5′-GAT TTA ATC TGT ATC AGG-3′). Protein expression was accomplished by growing and inducing 50 ml of cells as follows: 2 ml of LB broth containing 50 µg/ml ampicillin were inoculated with a single colony and grown overnight at 37°C with shaking. Subsequently, 50 ml of LB broth containing 50 µg·ml^−1^ ampicillin were inoculated with 1 ml of the overnight culture and grown until mid-log. The culture was then induced with IPTG to a final concentration of 1 mM and the culture grown at 37°C with shaking at 100 rpm for 5 h. The cells were then harvested by centrifugation at 3000 *g*, 10 min, 4°C, and the pellets stored at −80°C before proceeding to protein purification. Purification of the proteins was carried out in native conditions using the ProBond™ Purification System (Invitrogen, Carlsbad, CA, USA). Sodium dodecyl sulfate-polyacrylamide gel electrophoresis (SDS-PAGE) was used to examine the success of the purification.

Protein concentration was estimated using the Bradford procedure [23] employing BSA as the standard (Sigma, Dorset, UK). The enzyme sample (5 µl) was mixed with 250 µl of Bradford reagent in a microplate, the plate was shaken for 30 s and incubated at room temperature for 20 min. The formation of the blue-coloured Coomassie-Blue G-250 complex was then monitored at 595 nm on a PowerWave XS microplate reader (BioTek Instruments Inc., Potton, UK).

### Phylogenetic placement

Predicted protein sequences were aligned using the built-in ClustalW (default parameters), and a phylogenetic tree built using the Maximum Parsimony method with default parameters and 500 bootstrap replications with the MEGA5 software [24].

### Enzymatic assays

Enzyme activity was quantified on a temperature-controlled Powerwave XS microplate reader (BioTek Instruments Inc., Potton, UK) based on the level of ρ-nitrophenol released following the hydrolysis of ρ-nitrophenyl ester substrates by the enzyme [25], [26]. The production of ρ-nitrophenol was monitored in triplicate every minute for 10 min at 410 nm, and data were collected with the software Gen5 v1.10 (BioTek Instruments Inc., Potton, UK). Unless otherwise described, enzyme activity was measured by a standard assay at 39°C, with 1 mM ρ-nitrophenyl ester substrates in 50 mM morpholineethanesulfonic acid (MES, pH 6.5) containing 1% acetonitrile. The substrate used in standard conditions was ρ-nitrophenyl caprylate (C8) for alipA, alipBss and alipC. After pre-incubation for 3 min, the reaction was started by the addition of 2 µl of the eluted fraction of purified enzyme (∼0.4 mg·ml^−1^). Blank reactions were performed with every measurement to subtract appropriate values for nonenzymatic hydrolysis of the substrate. One unit of enzyme activity was defined as the amount of activity required to release 1 µmol of ρ-nitrophenol·min^−1^ from ρ-nitrophenyl ester.

### Substrate specificity

The following ρ-nitrophenyl esters with different chain length were purchased from Sigma-Aldrich (Dorset, UK) or TCI Europe (Zwijndrecht, Belgium) and used at 1 mM final concentration for assaying substrate specificities: ρ-nitrophenyl butyrate (C4), ρ-nitrophenyl caproate (C6), ρ-nitrophenyl caprylate (C8), ρ-nitrophenyl caprate (C10), ρ-nitrophenyl laurate (C12), ρ-nitrophenyl myristate (C14), ρ-nitrophenyl palmitate (C16) and ρ-nitrophenyl stearate (C18). The ρ-nitrophenyl ester substrates with C4 to C10 acyl chains were dissolved in acetonitrile at a concentration of 100 mM. ρ-nitrophenyl ester substrates with C12 to C18 acyl chains were dissolved in a 1∶4 mixture of acetonitrile and 2-propanol in order to solubilise the substrate, and reactions were performed with final concentrations of 1% acetonitrile and 4% 2-propanol.

### Effect of pH on enzyme activity

The effect of pH on the activity of the enzymes was examined across the pH range 3.5 to 10.0 using a wide-range pH buffer [27], containing 40 mM each of acetic acid, MES, N-(2-hydroxyethyl) piperazine-N′-ethanesulfonic acid (HEPES), N-[Tris(hydroxymethyl) methyl]-3-aminopropanesulfonic sodium salt (TAPS) and N-cyclohexyl-3-aminopropane sulfonic acid (CAPS). The pH was adjusted by adding 1 M HCl or 1 M NaOH as appropriate at 39°C. The specific activity of the enzyme was determined photometrically at 348 nm as it is the pH-independent isobestic wavelength of ρ-nitrophenoxide and ρ-nitrophenol [28].

### Effect of temperature on enzyme stability and thermostability

The effect of temperature on the activity of enzyme activity was examined across the range 25–70°C under standard assay conditions. The pH of the MES buffer was adjusted to 6.5 at respective temperatures. The thermostability of the enzymes was analysed by measuring the residual activity after incubating the enzyme (2 µl in 50 mM MES, pH 6.5) for 1 h at 50, 60 and 70°C.

### Effect of metal ions

The effect of metal ions on the activity of the enzymes were investigated by incubating the enzymes with various metal chloride salts (Na^+^, K^+^, NH_4_^+^, Mg^2+^, Ca^2+^, Mn^2+^, Zn^2+^, Co^2+^) at final concentrations of 5 mM in 50 mM MES (pH 6.5) for 30 min at room temperature. The remaining activity was then measured under standard assay conditions.

### Nucleotide sequence accession numbers

The draft genome was deposited with NCBI BioProject Accession: PRJNA187036 The nucleotide sequences of the genes reported here are available in the GenBank database under accession numbers KC579357–KC579359.

## Results

### Pyrosequencing results and identification of three lipolytic genes in the draft genome

**Figure 1:**
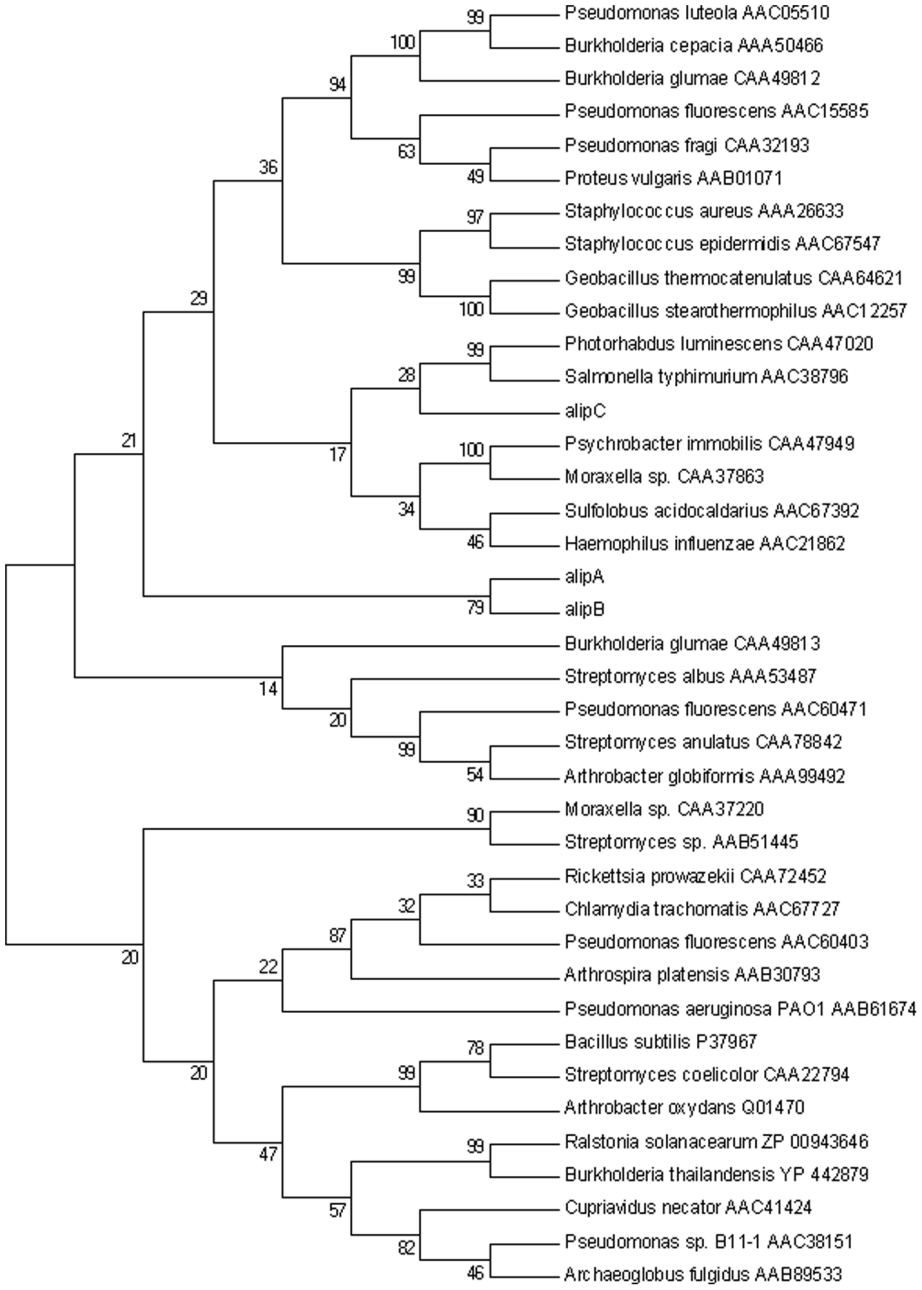
Maximum Parsimony tree of lipases. Protein sequences were aligned using the built-in ClustalW (default parameters), the tree was built using the Maximum Parsimony method with default parameters and 500 bootstrap replications.

Pyrosequencing generated 340,862 high quality reads with an average length of 425 bp, representing 144,706,594 bp of total information. These data represented 36× coverage for an estimated bacterial genome size of 4 Mbp. The assembly of the uncompleted draft genome resulted in 285 contigs with 2,830,874 bp total sequence information, comprising 247 large contigs (>5000 bp) with a total size of 2,816,384 bases. The RAST annotation identified 2,673 coding sequences and the G+C content was 43.3%. Copies of the 5ST and 23S rRNA genes (6 and 1 respectively) and 60 predicted tRNA genes were identified within the genome. There were 268 subsystems represented in the genome, however 63% of the predicted genes could not be assigned to a subsystem. Two genes annotated as “GDSL family lipolytic enzyme” and one gene annotated as “carboxylesterase” were named *alipA*, *alipB* and *alipC* respectively. Phylogenetic analysis indicated that alipA and alipB clustered with the GDSL/SGNH family II, and alipC clustered with lipolytic enzymes from family V (Figure 1).

### The lipase genes alipA, alipB and alipC identified as novel members of family II and V lipases

Gene length varied from 744 to 1,476 bp; and features of the encoded proteins are presented in Table 1. Tables 2 and 3 present the results of the BLASTN and BLASTP analysis of the identified putative lipase genes. Gene *alipA* matched with a gene coding for a lipolytic protein from *Selenomonas sputigena*, with 63% identity, whereas no homologous sequences were found for genes *alipB* and *alipC* in the Genbank database. However, the proteins were found to match with proteins from various *Veillonellaceae*, and the best hits were with GDSL lipolytic proteins from *Selenomonas* species for the proteins alipA and alipB (56 and 41% identity respectively) and with a lipase/esterase from *Mitsuokella multiacida* for alipC (51% identity). AlipC also shared 42% amino acid identity (e-value 8e^−50^) with a lipase from a rumen metagenome RlipE2 [29].

**Table 1:** Putative lipase/esterase genes identified from *Anaerovibrio lipolyticus* 5ST using the RAST annotation and features of the encoded proteins.

Gene	Length (bp)	Protein size (aa)	Protein molecular weight (kDa)	Theoretical isoelectric point
*alipA*	1476	492	56.27	5.64
*alipB*	1314	438	48.79	4.94
*alipC*	744	248	27.75	6.17

**Table 2:** Best matches obtained using BLASTN for the lipolytic genes identified in *Anaerovibrio lipolyticus* 5ST.

Contig	Gene	Best hit (accession number)	Nucleotide identities (%)	E-value
Contig00046	*alipA*	Lipolytic protein GDSL family (CP002637) from *Selenomonas sputigena* ATCC 35185	533/842 (63%)	2e^−13^
Contig00136	*alipB*	None	-	-
Contig00239	*alipC*	None	-	-

**Table 3:** Best matches using BLASTP for the predicted amino acid sequence of lipolytic genes identified in *Anaerovibrio lipolyticus* 5ST.

Protein	Putative function (accession number)	Most similar homolog (e-value)	Identity (overlapped aa)
alipA	Lipolytic protein GDSL family (AEC00120)	*Selenomonas sputigena* ATCC 35185 (0.0)	278/495 (56%)
alipB	GDSL-like protein (EFR39963)	*Selenomonas* sp. *oral* taxon 137 str. F0430 (5e^−108^)	169/411 (41%)
alipC	Putative esterase/lipase (EEX68534)	*Mitsuokella multiacida* DSM 20544 (3e^−80^)	126/249 (51%)

Domain analysis (Table S1) revealed that alipA and alipC did not contain a signal peptide; alipB contained a putative 24-residual signal peptide at the N-terminus. AlipA and alipB contained a unique SGNH/GDSL hydrolase superfamily domain (c|01053) at amino acid residues 306–482 and 242–413 respectively. No conserved domain could be identified on half of the protein sequences, on the N side. AlipC contained a COG1647 domain (esterase/lipase function prediction) at amino acid residues 1–244 and an esterase/lipase superfamily domain (c|12031) at amino acid residues 85–227.

**Figure 2:**
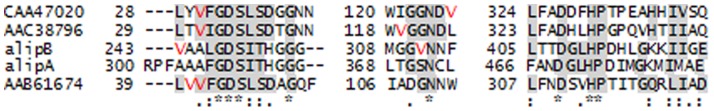
Conserved sequence motifs of alipA and alipB and lipolytic enzymes from the GDSL family. Alignment of Pfam conserved domains. The accession numbers of the aligned sequences are for the following organisms: CAA47020, triacylglycerol lipase from *Photorhabdus luminescens*; AAC38796, outer membrane esterase from *Salmonella typhimurium*; AAB61674, lipase/esterase from *Pseudomonas aeruginosa* PAO1. Conserved motifs are highlighted. The possible catalytic triad (Serine (S), Aspartic acid (D), Histidine (H)) is shown at the top of the alignment whenever necessary.

**Figure 3:**
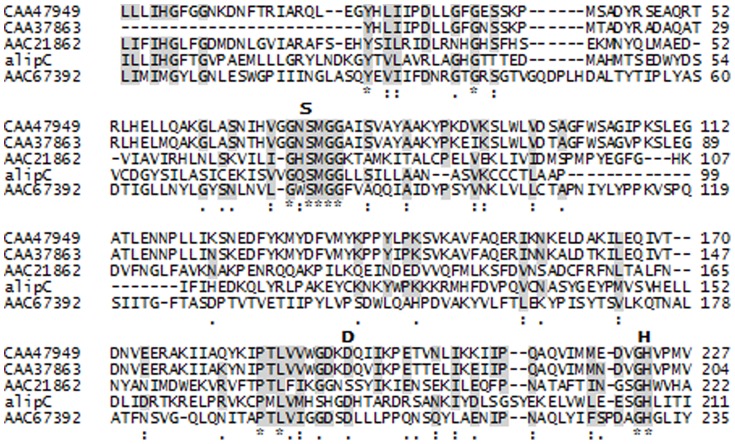
Conserved sequence motifs of alipC and lipolytic enzymes from family V. Alignment of Pfam conserved domains. The accession numbers of the aligned sequences are for the following organisms: CAA47949, triacylglycerol lipase from *Psychrobacter immobilis*; CAA37863, triacylglycerol lipase from *Moraxella* sp.; AAC21862, putative esterase/lipase from *Haemophilus influenzae*; AAC67392, lipolytic enzyme from *Sulfolobus acidocaldarius*. Conserved motifs are highlighted. The possible catalytic triad (Serine (S), Aspartic acid (D), Histidine (H)) is shown at the top of the alignment whenever necessary.

The proteins alipA and alipB contained, respectively, the lipase-conserved catalytic triad residues Asp466/Asp405 and His469/His408 and the catalytic nucleophile Ser309/Ser249 in a GDS(L) motif (Figure 2). These indicated that alipA and alipB were related to enzymes from family II as defined by Arpigny and Jaeger [30]. The protein alipC contained the catalytic triad Asp194, His224 and Ser90 in the pentapeptide motif GQSMG, and multiple amino acid alignments indicated alipC would be a new member of family V (Figure 3).

### Expression and purification of alipA, alipB and alipC

**Figure 4:**
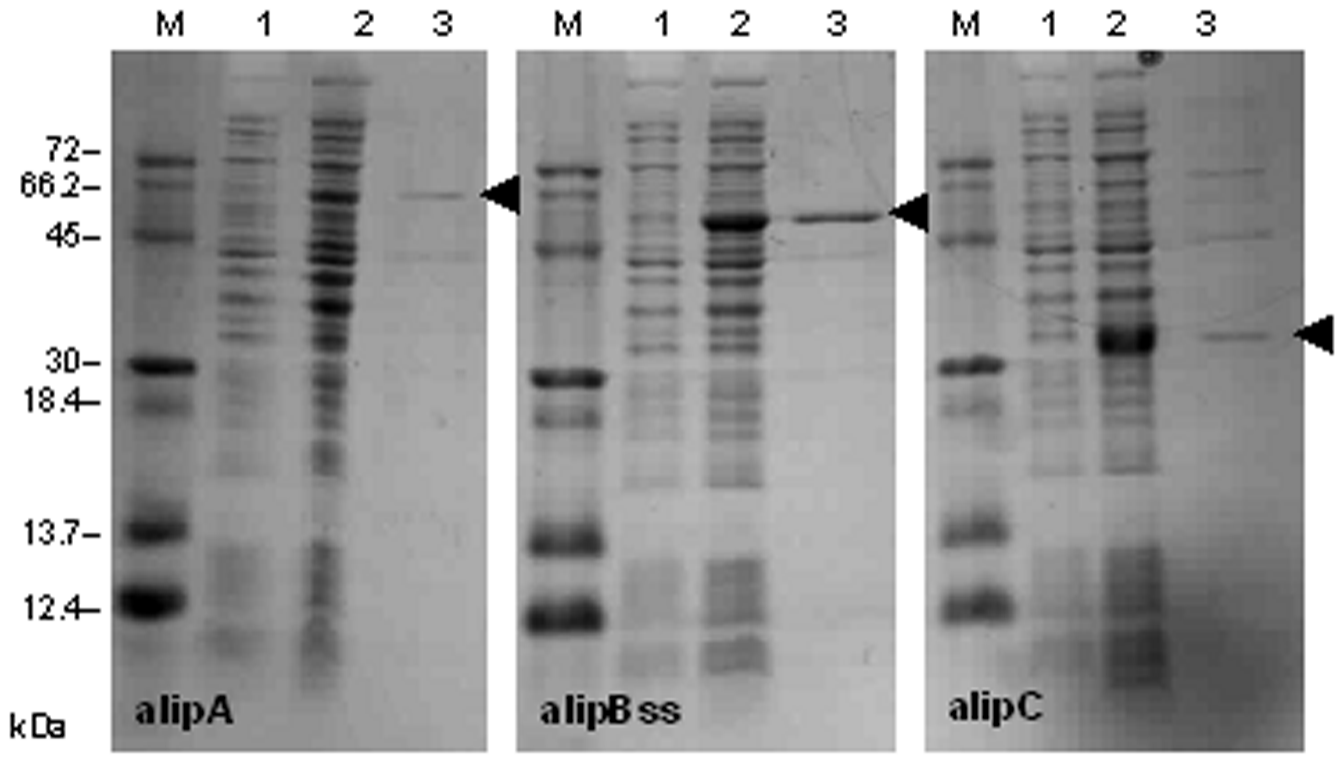
Analysis of the proteins expressed in *Escherichia coli* TOP10 cells following purification on a 15% denaturing polyacrylamide gel. Lanes M, protein standards; 1, before induction with 1 mM IPTG; 2, after cells were induced with IPTG and grown at 37°C for 5 h; 3, recombinant protein purified under native conditions with the ProBond™ purification system. Arrows designate the positions of the proteins on the gels.

In order to investigate the biochemical properties of the enzymes, they were expressed in the pTrcHis TOPO vector in *Escherichia coli*. AlipB without its signal sequence (alipBss) was chosen as alipB was either not expressed, degraded or insoluble during expression as it could not be detected on SDS-PAGE (data not shown). Figure 4 illustrates the purification process, with the total lysates of *E. coli* TOP10 expressing the recombinant proteins before and after induction with 1 mM IPTG, and the purified fraction after elution from the nickel resin. The purification protocol routinely yielded 0.2 to 0.4 mg·ml^−1^ of purified protein from 50 ml cultures grown for 5 h after induction.

### Substrate specificity

To examine substrate specificity, activity was tested against various ρ-nitrophenyl esters with different acyl chain lengths. The results under standard assay conditions of pH 6.5 and 39°C are presented in Table 4. AlipA and alipBss showed a narrow chain length specificity, with the highest specific activity against ρ-nitrophenyl laurate (640 U·mg^−1^) and myristate (157 U·mg^−1^) respectively, and lower specific activity against ρ-nitrophenyl caproate (33 and 43 U·mg^−1^ respectively). AlipC showed a broader range of activity with higher specific activities against short to medium acyl chain length: the activities were 187 U·mg^−1^ against ρ-nitrophenyl butyrate, 270 U·mg^−1^ against ρ-nitrophenyl caprylate, 118 and 242 U·mg^−1^ against ρ-nitrophenyl laurate.

**Table 4:** Activity of lipases isolated from *Anaerovibrio lipolyticus* 5ST against different substrates.

Substrate	Specific activity (U·mg^−1^ protein)		
	alipA	alipBss	alipC
**pNP-acylesters**			
Butyrate (C4)	139.4±17.4	91.8±22.9	186.7±41.5
Caproate (C6)	73.8±19.0	48.6±11.5	76.0±48.9
Caprylate (C8)	98.4±17.4	59.4±21.8	269.6±71.7
Caprate (C10)	32.8±9.5	43.2±11.4	117.5±5.3
Laurate (C12)	639.6±24.0	97.2±24.7	242.0±38.4
Myristate (C14)	172.2±17.4	156.6±11.5	193.6±34.2
Palmitate (C16)	123.0±19.1	64.8±27.6	83.0±37.5
Stearate (C18)	73.8±17.3	ND	34.6±20.8

ND. Not detected.

### Effect of pH and temperature on enzyme activity

**Figure 5:**
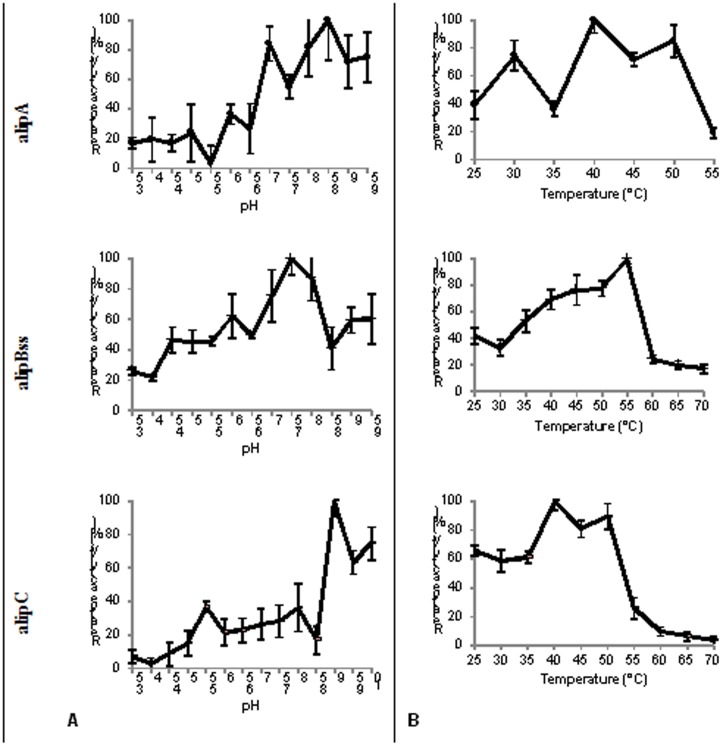
The effect of pH (A) and temperature (B) on the activity of proteins alipA, alipBss and alipC. The pH assays were determined against ρ-nitrophenyl caprylate (C8) at a constant temperature of 39°C in a wide-range pH buffer set at the indicated pH values. Temperature assays were determined against the same substrate at a constant pH of 6.5 in 50 mM MES.

The effects of pH and temperature on the activity of the enzymes were determined (Figure 5). AlipA and alipC had maximal activity at pH 8.5 and 9.0 respectively, and presented >50% activity in alkaline pH ranges, respectively 7.5–9.5 and 9.0–10.0. AlipBss showed >50% of maximum activity in the pH range 6.0–8.0, with maximal activity at pH 7.5. The optimum temperatures were determined as 40°C (alipA, alipC) and 55°C (alipBss). The temperature range where the enzyme retained more than 50% activity was 40–50°C for alipA, 35–55°C for alipBss, and 35–50°C for alipC. The temperature stability of the proteins was examined by measuring its residual activity after incubating the purified enzymes for 1 h at 50, 60 or 70°C (Table 5) and thus represent both temperature stability including the protein unfolding and refolding potential of the proteins following thermal shock. The proteins alipBss and alipC appeared to be temperature sensitive as less than 50% of activity was measured after 1 h incubation at 50°C. Activities ranged from 8 to 45% after incubating at 60 or 70°C. AlipA appeared to have some thermostability: it retained around 50% activity after incubation at 60 and 70°C.

**Table 5:** Relative activity of lipases cloned from *Anaerovibrio lipolyticus* 5ST after incubation for one hour at 50, 60 or 70°C.

	Relative activity (%)		
Temperature of incubation			
	alipA	alipBss	alipC
40°C	100.0	100.0	100.0
50°C	16.8±9.8	33.6±11.0	32.8±12.4
60°C	45.3±12.5	24.0±6.1	8.2±5.8
70°C	49.2±8.4	15.6±6.5	9.8±3.2

The enzymes were incubated 1 h at 50, 60 and 70°C in 50 mM MES buffer (pH 6.5); the residual activities were measured with a standard assay against ρ-nitrophenyl caprylate (C8). The activity of the enzyme at 40°C was defined as 100%. Control activities at 40°C were 794, 564 and 793 U·mg^−1^ protein for alipA, alipBss and alipC respectively).

### Effect of ions on enzyme activity

The effects of different metal cations at a concentration of 5 mM on the activities of the enzyme was assayed, the results are presented in Table 6. Ca^2+^, Mn^2+^ and Co^2+^ inhibited alipA activity(<68% activity), K^+^ and NH_4_^+^ did not modify the activity (98–99% activity), while Na^+^, Mg^2+^ and Zn^2+^ activated alipA. AlipBss activity was strongly inhibited by Ca^2+^ and Mg^2+^ (residual activities, 33 and 56% respectively) and moderately inhibited by Na^+^, K^+^ and NH_4_^+^ (residual activities, 72, 67 and 69% respectively), whereas Mn^2+^, Zn^2+^ and Co^2+^ activated alipBss (residual activities, 130, 107, and 103% respectively). Zn^2+^ strongly inhibited alipC activity (17% residual activity); NH_4_^+^, Mg^2+^ and Co^2+^ had more moderate inhibitory effects (residual activities from 74 to 81%). K^+^ had no effect, while Na^+^, Ca^2+^ and Mn^2+^ activated alipC (residual activities, 119, 185, and 141% respectively).

**Table 6:** Relative activity of lipases cloned from *Anaerovibrio lipolyticus* 5ST after incubation for 30 min with different metal ions.

Relative activity (%)			
Ions	alipA	alipBss	alipC
unincubated	100.0	100.0	100.0
Na^+^	107.3±2.5	71.6±5.7	119.4 ±5.4
K^+^	98.3±4.0	67.1±1.6	100.5±3.6
NH_4_^+^	95.3±5.2	69.3±5.2	73.9±1.5
Mg^2+^	113.2±2.2	55.9±1.9	73.9±1.2
Ca^2+^	32.8±1.1	35.8±8.4	184.8±1.3
Mn^2+^	68.5±2.3	129.7±20.2	141.1±2.0
Zn^2+^	107.3±5.0	107.4±9.4	16.8±1.5
Co^2+^	56.6±1.9	102.9±1.3	80.7±2.0

The enzymes were incubated 30 min in 50 mM MES buffer (pH 6.5) with the metal ions at 5 mM final concentration; the residual activities were measured with a standard assay against ρ-nitrophenyl caprylate (C8). The activity of the enzyme not incubated with ions was defined as 100%. Control activities (inincubated) were 337, 296 and 270 U·mg^−1^ protein for alipA, alipBss and alipC respectively).

## Discussion

Next-generation sequencing has provided scientists with quick and increasingly affordable methods to access genomic data. The 454 technology has been used successfully in a number of studies to establish draft and complete bacterial genomes and establish their gene catalogues, for example for the bacteria *Leuconostoc argentinum*, *Lactobacillus animalis* [31], [32], *Staphylococcus epidermidis* A487 [33], *Acinetobacter baumannii* [34]; and, combined with other sequencing platforms, the genomes of the rumen bacteria *Megasphaera elsdenii* [35], *Ruminococcus albus* [36] and *Fibrobacter succinogenes* [37].

The genus *Anaerovibrio* currently includes a single species, *A. lipolyticus*. The 454 technology was used in this study to establish the draft genome of *A. lipolyticus* 5ST, and identify putative lipase genes.

The draft genome was annotated using the Rapid Annotation using Subsystem Technology (RAST) server [16]. However, the accuracy of the annotation also relies on the automated pipeline used [38], some predicted genes could be dissimilar to anything in the reference databases as they could have evolved extensively, represent uncharacterized sequences, or be misidentified [39]. Reference databases and computational methods constituting annotation pipelines are constantly developed, and there is hence a need to reprocess genome annotations on a regular basis to improve their quality and completeness [39], [40]. As it was not the primary objective in this study, the genomic sequence of the bacterium remains as draft. However it would be valuable to complete the draft sequence, and subsequently annotate the complete genome, as the presence of other lipolytic genes may have been overlooked. In addition a closed genome would be useful to better understand *A. lipolyticus*'s role in the rumen and to share its genome in public databases for future use in molecular studies.

*AlipA*, *alipB* and *alipC* exhibited very low nucleotide as well as amino acid sequence similarity to previously available sequences, except for a rather low match with some amino acid sequences from the Veillonellaceae genera *Selenomas*, *Mitsuokella* and *Centipeda*. It is therefore proposed that they represent novel esterases/lipases, and that they have not been isolated yet from previous metagenomic studies in the rumen [29], [41].

AlipA and alipB exhibited the distinct GDSL sequence motif located at the N-terminal part and Ser, Asp and His residues as part of the active site. They also contained a SGNH hydrolase superfamily domain, thus classifying them as belonging to the subfamily of GDSL/SGNH enzymes [42], [43], [44]. The GDSL family of enzymes was first identified by Upton and Buckley [42], these enzymes contain a GDS(L) motif located in the N-terminal part of the protein instead of the conserved lipase motif GXSXG [30]. The SGNH hydrolase subfamily was proposed from the crystal structures of *Aspergillus aculeatus* rhamnogalacturonan acetylesterase, *Streptomyces scabies* and influenza C virus esterases and an acetylhydrolase isolated from *Bos taurus* [45], [46]; where four amino acids were found to be essential for catalysis, namely serine, glycine, asparagine, and histidine. The catalytic serine is located in a GDS(L) motif, while the Gly and Asn serve as proton donors to the oxyanion hole, and the His helps increasing the nucleophilicity of the Ser by deprotonating its hydroxyl group [45]. However sequence analysis showed that only the second half of alipA and alipB amino acid sequences were predicted for esterase/lipase function, as the conserved domains were present on amino acid residues 306–482 and 242–413 on the native sequence of alipA (492 aa total) and alipB (438 aa total) respectively. No conserved domain or homologies could be detected on the first part of the protein sequences; thus further studies would be needed to assess whether these parts of the proteins would contain other catalytic domains or serve protein structural functions such as autotransport. For example, GDSL hydrolases from *Pseudomonas aeruginosa*, *S. typhimurium* and *Photobacterium luminescens* harbour a C-terminus domain encompassing approximately one third of the entire protein. This domain is composed of 12 β-sheets which form a β-barrel inserted into the bacterial outer membrane and forms a structure similar to porines [47], [48], [49]. The N-terminal part of the protein carrying the catalytic activity is exported through the β-barrel and often cleaved off after translocation [50], [51], [52], [53], [54], [55]. Enzymes belonging to the GDSL family were also described as exhibiting broad substrate specificity, due to a flexible active site that changes conformation with the binding of different substrates [43], and regioselectivity. In contrast, alipA had a very high specific activity against ρ-nitrophenyl laurate, and alipBss against ρ-nitrophenyl myristate. Assessment of their activity towards a broader range of substrate would be needed to further characterize these enzymes.

Analysis of the alipC-encoded protein showed that the protein belongs to the serine hydrolase group and has common features to basic lipases [30], as well as the conserved sequence motifs of family V bacterial lipases in its deduced amino acid sequence. It is therefore proposed that alipC could be grouped in this family. Lipases from family V have predicted molecular masses averaging 30 kDa [44] ; likewise alipC was a small, single-domain protein with a molecular mass of 27.75 kDa and a pI of 6.17. Comparison of the biochemical properties of alipC with those of other lipases is however difficult since very little information about the family V of lipases is available. Members of family V are proteins sharing significant homology with other bacterial enzymes such as epoxide hydrolases, dehalogenases and haloperoxidases, which also possess the α/β hydrolase fold [30], [44]. Only four enzymes from this family have been cloned and characterized to date: the carboxylesterase Est2 from *Acetobacter pasteurianus* showing maximum activity at 78°C and pH 7.8 [56], [57], the carboxylesterase EstV from *Helicobacter pylori* with an optimum temperature of 50°C and pH of 10 [58], the lipase Lip1 isolated from the thermophilic bacterium *Fervidobacterium changbaicum* showing maximum activity at 78°C and pH 7.8 [27] and the lipase RlipE2 from a metagenomic library of bovine rumen with maximum activity at 30°C and pH 7.5 [29]. Both Est2 and EstV presented typical characteristics of carboxylesterases, with EstV showing preference for short chain length ρ-nitrophenyl esters (C2–C6) and triglycerides (C4) (Ruiz *et al.*, 2007). Est2 hydrolyzed preferentially triglycerides with even shorter chain lengths like triacetin (C2) and tripropionin (C3) [57]. The enzyme FCLip1 exhibited preference for medium chain length ρ-nitrophenyl esters (C10) and tricaprylin (C8) [27], while RlipE2 showed high hydrolytic activity against longer chain ester substrates (C12, C16, C18) and triolein (C18) [29]. The characterization of alipC therefore gives new insights into lipase family V.

*A. lipolyticus* 5ST lipase activity was investigated by methods available in the 1970s [8], [15]. An extracellular lipase being associated with cell surface or extracellular membranous structures was described. Activity was measured in the pH range 6.6–7.8 and temperature range 15–35°C. Optimum activity was observed at pH 7.4 and temperature 20 to 22°C, activity was enhanced by CaCl_2_ and BaCl_2_ while ZnCl_2_ and HgCl_2_ were inhibitory. Diglycerides were hydrolyzed more rapidly than triglycerides. AlipA, alipB and alipC do not match this description, though enzyme activities with different buffers and substrates cannot be directly compared [59]. Only alipB had a signal peptide suggesting it might be secreted from the cell and its activity was somewhat inhibited by Ca^2+^ and slightly enhanced by Zn^2+^. AlipC activity was enhanced by Ca^2+^ and strongly inhibited by Zn^2+^, but maximal activity was observed at pH 9. Fay and colleagues [60] also observed that pure cultures of *A. lipolyticus* 5ST could not efficiently catalyze ρ-nitrophenyl palmitate and concluded this substrate was more likely to indicate esterase activity rather than lipase activity.

So far, only six pure cultures of obligate anaerobic and lipolytic bacteria have been isolated from the rumen of sheep, cattle and deer [1], [10], [61], [62], [63], [64] (. It was estimated that *A. lipolyticus* alone could account for the total rates of esterified fatty acid production in the rumen, based on lipid hydrolysis rates and population density studies [65]. Three lipase/esterase genes from the draft genome of *A. lipolyticus* 5ST were isolated, and their characterization is an important step in increasing our knowledge on the lipase activity within the rumen.

## Supporting Information

Table S1 **Conserved domains and predicted signal sequences in the proteins alipA, alipB and alipC.**
(DOCX)

Table S2 **Primers used for amplification of the lipolytic genes in** ***Anaerovibrio lipolytica*** **5S.**
(DOCX)
