# Supplementary material for: Identification and Characterization of Three Novel Lipases Belonging to Families II and V from Anaerovibrio lipolyticus 5ST
Source: PLoS One. 2013 Aug 12;8(8):e69076. doi: 10.1371/journal.pone.0069076 (PMC3741291; doi:10.1371/journal.pone.0069076)
Supplement: Table S1 — Conserved domains and predicted signal sequences in the proteins alipA, alipB and alipC. (DOCX) [file pone.0069076.s001.docx]

**Table S1. Conserved domains and predicted signal sequences in the proteins alipA, alipB and alipC**

| **Protein** | **Conserved domain** | **Positions on native protein** | **E-value** | **Predicted signal sequence (cleavage position)** |
| --- | --- | --- | --- | --- |
| alipA | SGNH hydrolase superfamily (c\|01053) | 306-482 | 1.47e^-18^ | No |
|  | Active site | 309, 343, 372, 466, 469 |  |  |
|  | Catalytic triad | 309, 466,469 |  |  |
|  | Oxyanion hole | 309, 343, 372, |  |  |
| alipB | SGNH hydrolase superfamily (c\|01053) | 242-413 | 1.95e^-21^ | VVA-ED (24-25) |
|  | Active site | 249, 283, 312, 405,408 |  |  |
|  | Catalytic triad | 249, 405, 408 |  |  |
|  | Oxyanion hole | 249, 283, 312, |  |  |
| alipC | COG1647 (esterase/lipase) | 1-244 | 1.41e^-63^ | No |
|  | Esterase/lipase superfamily (c\|12031) | 85-227 | 1.28e^-03^ |  |
