# Supplementary material for: Identification and Characterization of Three Novel Lipases Belonging to Families II and V from Anaerovibrio lipolyticus 5ST
Source: PLoS One. 2013 Aug 12;8(8):e69076. doi: 10.1371/journal.pone.0069076 (PMC3741291; doi:10.1371/journal.pone.0069076)
Supplement: Table S2 — Primers used for amplification of the lipolytic genes in Anaerovibrio lipolytica 5S. (DOCX) [file pone.0069076.s002.docx]

**Table S2. Primers used for amplification of the lipolytic genes in *Anaerovibrio lipolytica* 5S**

| **Gene amplified** | **Primer** | **Sequence (5'-3')** | **Expected size of product (bp)** |
| --- | --- | --- | --- |
| *alipA* | alipA F | acc atg gat tgg acc cgt tat | 1482 |
|  | alipA R | tgc tta ttg ctt aaa tac ttc ctt gtg |  |
| *alipB* | alipB F | ggg aga gat aac atg aag ttt tcc | 1344 / 1263(ss) |
|  | alipB ssF | gca gag gat att aat act gag |  |
|  | alipB R | ttc gtg aga cag ctt ttt tta |  |
| *alipC* | alipC F | acc agc atg att att aac ggc | 855 |
|  | alipC R | CAC CGG CTC TCT ATG TGT TAT |  |

F, forward primer; ssF, forward primer with predicted signal sequence removed; R, reverse primer
